# Tobacco use and outcome in radical prostatectomy patients

**DOI:** 10.1002/cam4.1041

**Published:** 2017-03-20

**Authors:** Alexandra Curtis, Rochelle Payne Ondracek, Christine Murekeyisoni, Eric Kauffman, James Mohler, James Marshall

**Affiliations:** ^1^ Department of BiostatisticsUniversity of IowaIowa CityIowa; ^2^ Department of Cancer PreventionRoswell Park Cancer InstituteBuffaloNew York; ^3^ Department of BiostatisticsUniversity at BuffaloBuffaloNew York; ^4^ Department of UrologyRoswell Park Cancer InstituteBuffaloNew York; ^5^ Center for ImmunotherapyRoswell Park Cancer InstituteBuffaloNew York; ^6^ Department of Urology and Department of Cancer GeneticsRoswell Park Cancer InstituteBuffaloNew York; ^7^ Department of UrologyState University of New York at BuffaloBuffaloNew York

**Keywords:** Cancer, history, outcomes, prostate, smoking

## Abstract

Cigarette smoking has been consistently associated with increased risk of overall mortality, but the importance of smoking for patients with prostate cancer (CaP) who are candidates for curative radical prostatectomy (RP) has received less attention. This retrospectively designed cohort study investigated the association of smoking history at RP with subsequent CaP treatment outcomes and overall mortality. A total of 1981 patients who underwent RP at Roswell Park Cancer Institute (RPCI) between 1993 and 2014 were studied. Smoking history was considered as a risk factor for overall mortality as well as for currently accepted CaP treatment outcomes (biochemical failure, treatment failure, distant metastasis, and disease‐specific mortality). The associations of smoking status with these outcomes were tested by Cox proportional hazard analyses. A total of 153 (8%) patients died during follow‐up. Current smoking at diagnosis was a statistically significant predictor of overall mortality after RP (current smokers vs. former and never smokers, hazards ratio 2.07, 95% confidence interval [CI]: 1.36–3.14). This association persisted for overall mortality at 3, 5, and 10 years (odds ratios 2.07 [95% CI: 1.36–3.15], 2.05 [95% CI: 1.35–3.12], and 1.8 [95% CI: 1.18–2.74], respectively). Smoking was not associated with biochemical failure, treatment failure, distant metastasis, or CaP‐specific mortality, and the association of smoking with overall mortality did not appear to be functionally related to treatment or biochemical failure, or to distant metastasis. Smoking is a non‐negligible risk factor for death among CaP patients who undergo RP; patients who smoke are far more likely to die of causes other than CaP.

## Introduction

CaP, the most commonly diagnosed noncutaneous cancer in men, accounts for 13.3% of new cancer cases and 4.7% of cancer deaths in the United States each year 1. Since the implementation of prostate‐specific antigen (PSA) screening for early detection, many patients diagnosed with CaP die of other diseases, particularly cardiovascular disease, which has been unequivocally linked to smoking 2.

Although smoking has not been shown to increase CaP risk, there is concern that smoking may increase CaP aggressiveness and risk of CaP‐specific mortality 3. This study tested the association of smoking history at RP (current, former, never), among well‐annotated and carefully followed RP patients, with multiple, currently recognized measures of CaP‐specific outcomes and overall mortality.

## Materials and Methods

### Description of participants

After approval from the RPCI IRB, information was abstracted from the medical records of 1981 patients who had undergone RP at RPCI between 1993 and 2014. The annotating data included clinical stage and Gleason score, RP date, pathologic stage and Gleason score, surgical margin and lymph node status, serum PSA values, the date and type of CaP treatments before or after RP, and smoking history. Table 1 summarizes prognostic factors considered in this analysis. Pathology information (tumor stage, Gleason score, margin status) was determined by RPCI genitourinary pathologists. Patients were staged according to 2002 TNM guidelines. Patients were followed until 2014, using patient, outside urologist, Roswell Park registry, and primary care practitioner correspondence to track outcomes and overall survival. (The Roswell Park Registry is linked to the New York State Tumor Registry.) Patients lost to follow‐up were censored after the date of last contact. Metastatic disease and diagnosis date, death and cause of death (CaP‐specific or other) were extracted from medical records. The mean and median RPCI clinic follow‐up for this cohort were 4.4 and 3.6 years, respectively, with range 0–20.8 years. The mean and median survival follow‐up (which made use of outside correspondence) were 5.9 and 4.9 years, respectively, with a range 0–20.7 years.

**Table 1:** Characteristics of never, former, and current smokers among 1924 prostatectomy patients

Characteristic	Never		Former		Current		*P*
*N* (%)	951 (49.4)		681 (35.4)		292 (15.2)		
Age at RP, mean (median)	60 (60)		62 (62)		59 (59)		<0.001
BMI (kg/m^2^), mean (median)	29.2 (28.4)		29.6 (28.9)		28.2 (27.7)		<0.001
Race							
White	854	92.1%	634	94.4%	242	84.9%	<0.001
Black	73	7.9%	38	5.7%	43	15.1%	
Unknown/other	24		9		7		
Pre‐RP recurrence risk category							
Low	354	37.3%	246	36.4%	93	32.0%	0.36
Intermediate	418	44.1%	317	47.0%	147	50.5%	
High	162	17.1%	103	15.3%	44	15.1%	
Very high	14	1.5%	9	1.3%	7	2.4%	
Unknown	3		6		1		
Diagnostic PSA (ng/mL)							
<4	142	15.0%	99	14.6%	30	10.3%	0.04
4 to <10	638	67.6%	466	68.6%	189	65.2%	
10 to <20	111	11.8%	86	12.7%	51	17.6%	
≥20	53	5.6%	28	4.1%	20	6.9%	
Unknown	7		2		2		
NADT							
No	886	93.6%	621	91.6%	263	90.4%	0.15
Yes	62	6.6%	57	8.4%	28	9.6%	
Unknown	3		3		1		
Pathologic Gleason sum							
<7	306	32.3%	207	30.4%	70	24.1%	0.12
=7 (3 + 4)	385	40.7%	296	43.5%	132	45.4%	
=7 (4 + 3)	161	17.0%	109	16.0%	62	21.3%	
>7	95	10.0%	69	10.1%	27	9.3%	
Unknown	4		0		1		
Pathologic stage							
T2a/T2b/T2x	101	10.6%	91	13.4%	36	12.3%	0.28
T2c	526	55.3%	369	54.2%	149	51.0%	
T3a	212	22.3%	161	23.6%	79	27.1%	
T3b	85	8.9%	48	7.1%	20	6.9%	
T4	27	2.8%	12	1.8%	8	2.7%	
Surgical margin status							
Positive	241	25.3%	162	23.9%	94	32.2%	0.02
Negative	710	74.7%	518	76.2%	198	67.8%	
Indeterminate	0		1		0		
Lymph node status at RP							
Positive	23	2.5%	9	1.4%	12	4.3%	0.02
Negative	910	97.5%	658	98.7%	268	95.7%	
Unknown	18		14		12		
Biochemical failure							
Yes	189	21.0%	159	25.0%	74	27.0%	0.06
No	710	79.0%	476	75.0%	200	73.0%	
Unknown	52		46		18		
Treatment failure							
Yes	232	25.8%	183	28.8%	83	30.4%	0.22
No	667	74.3%	452	71.2%	190	69.6%	
Unknown	52		46		19		
Distant metastasis							
Yes	34	3.6%	24	3.5%	12	4.1%	0.86
No	917	96.4%	657	96.5%	280	95.9%	
Overall mortality							
Yes	42	4.5%	58	8.7%	38	13.2%	<.001
No	892	95.5%	612	91.3%	251	86.9%	
Unknown	17		11		3		

A time‐dependent response to tobacco use was expected based on the results of earlier studies 4, 5. To address this, smoking status was classified as never, former, or current. Those classified as “never smokers” reported having smoked less than 1 pack year or having smoked for less than 1 year. “Former smokers” had smoked ≥1 year, but had quit ≥6 months before RP. (Five smokers with extremely low levels of smoking were reclassified as never smokers.) “Current smokers” had smoked ≥1 year, but either continued smoking or had quit <6 months before RP. Fifty‐seven (3%) patients with unknown smoking status or missing prognostic factors were excluded from analysis.

Pack years smoked was available for approximately 70% (684 of 973) of the former and current smokers. In a secondary analysis, these pack year data were used to further classify former and current smokers into “light” and “heavy” categories based on whether pack years were less than or more than the median of 25 pack years. “Light former smokers” had ≤25 pack years smoked, and “heavy former smokers” had >25 pack years smoked. The “light” and “heavy” classifications for current smokers used the same definitions.

Patient outcomes were defined by five oncological measures: (1) NCCN‐defined biochemical failure^6^ (biochemical failure defined as either persistent disease—failure of PSA to fall to undetectable levels after RP—or biochemical recurrence—undetectable PSA after RP, followed by a detectable PSA increasing on 2 or more determinations); (2) treatment failure, defined either as biochemical failure or treatment using radiation or androgen deprivation after RP; (3) distant metastasis (confirmed by either biopsy or radiology); (4) CaP‐specific mortality; and (5) overall mortality, defined as death from any cause, including unknown causes.

### Statistics

Statistical analyses were conducted using Stata 11. Prognostic factors were tested for association with smoking status using the chi‐square test for independence for categorical variables or the Mann–Whitney *U* test or Kruskall–Wallis ANOVA for continuous variables. Cox proportional hazards models at 3, 5, and 10 years, and for the entire period of observation, were used to determine whether the occurrence of these five outcomes (NCCN biochemical failure 6, treatment failure, distant metastasis, disease‐specific mortality, and overall mortality) in current smokers differed from those of former or never smokers. Differences between current smokers compared to former and never smokers together were also considered. All analyses were adjusted for characteristics known to be associated with CaP aggressiveness: pathologic Gleason sum, diagnostic PSA, surgical margin status, lymph node status, and pre‐RP risk recurrence category, as well as parameters shown to be significantly different among the smoking groups in this study: BMI, race, and age.

## Results

Most members of the cohort were overweight (45%) or obese (37%) (as defined by BMI >25 or >30, respectively), self‐identified as Caucasian American (90%), and had a low (36%) or intermediate (45%) NCCN recurrence risk, based on biopsy and PSA characteristics 6. Most patients had diagnostic PSA values between 4 and 10 ng/mL (67%). Few patients (8%) received NADT, and most had favorable pathologic characteristics: pathologic Gleason sum <7 (31%) or 3 + 4 (42%), ≤T2c pathologic tumor stage (66%), negative surgical margins (74%), and negative lymph nodes (95%). Never smokers comprised 49% of the cohort, former smokers accounted for 35%. Current smokers represented 15% of patients. Smoking status was unknown in 57 (3%) patients; these patients were excluded from analysis. Pack years was known for 684 (70%) of the 973 former or current smokers (overall data not shown).

Table 1 shows the association of smoking status with prognostic factors. Current smokers were younger, less obese, and more likely to be African American than never or former smokers. The diagnostic PSA values of current smokers were significantly higher than those of never or former smokers. Current smokers were more likely to have positive surgical margins, and were more likely to have positive lymph nodes at RP. The unadjusted overall mortality rate for current smokers was 13.2% compared to 8.7% for former smokers and 4.5% for never smokers (*P *<* *0.001).

Table 2 shows the associations of likely control variables with mortality. The associations of race, diagnostic PSA, surgical margin status, and lymph node status with mortality were negligible. The pre‐RP recurrence risk category was associated with a dose–response pattern of increasing risk, and the very high category with a hazard ratio of 3.43. The pathologic Gleason sum and tumor stage were associated with increased risk as well.

**Table 2:** Mortality risk factors among 1924 prostatectomy patients

	HR	*P*
Age at RP (continuous)	1.08	0
BMI	1.01	0.44
Race		
European American	Ref	
African American	0.94	0.89
Other	Could not estimate	
Pre‐RP recurrence risk category		
Low	Ref	
Intermediate	1.58	0.02
High	2.05	0.002
Very high	3.43	0.02
Diagnostic PSA		
<4	Ref	
4–<10	0.8	0.39
10–<20	1.33	0.33
≥20	1.52	0.23
Pathologic Gleason sum		
<7	Ref	
=7 (3 + 4)	1.1	0.66
=7 (4 + 3)	1.98	0.01
>7	3.22	0
Pathologic tumor stage		
T2a/T2b/T2x	Ref	
T2c	1.32	0.37
T3a	1.68	0.13
T3b	4.19	0
T4	1.33	0.72
Surgical margin status		
Negative	Ref	
Positive	1.08	0.70
Lymph node status		
Negative	Ref	
Positive	1.95	0.11

Table 2 shows the overall hazard ratios of each of the outcome measures (biochemical failure, treatment failure, distant metastasis, disease‐specific mortality, and overall mortality) by smoking status, using proportional hazards analysis for the entire period of follow‐up. The results are adjusted for the factors noted in the methods, Tables 1, 2, including age, BMI, race, pre‐RP recurrence risk, diagnostic PSA, pathologic Gleason sum and tumor stage, surgical margin, and lymph node status. The mortality analysis for the entire follow‐up period is upon data from 274 current smokers, 635 former smokers, and 934 never smokers, 1843 subjects in total. The associations of smoking status with biochemical failure, treatment failure, and distant metastasis for the entire follow‐up period were negligible, as were the hazards analysis associations of smoking status with these outcomes at 3, 5, and 10 years after RP. Thus, the only analyses presented for outcomes at 3, 5, and 10 years are for PCa‐specific mortality, non‐PCa mortality, and total mortality: these mortality analyses, as Table 3 shows, are based, respectively, on total sample sizes of 189 + 482 + 628 + = 1299; 147 + 375 + 430 = 952; and 80 + 177 + 163 = 420 subjects. These percentages amount to 70%, 52%, and 23% of total subjects. Current smokers had a significantly higher hazards ratio for overall mortality than former and never smokers, and the hazards ratios comparing never smokers to former and never smokers combined were statistically significant.

**Table 3:** Smoking status^a^ and mortality among 1924 prostatectomy patients

	Current versus Former			Current versus Never			Current versus Former and never		
	HR	*P*	95% CI	HR	*P*	95% CI	HR	*P*	95% CI
Cox proportional hazards (422 biochemical failure, 498 treatment failure, 70 distant metastasis, 23 PrCa deaths, 138 deaths from any cause)									
Biochemical failure C:274, F:635, N:899	0.8	0.35	0.50–1.28	1.13	0.33	0.89–1.43	0.99	0.97	0.64–1.54
Treatment failure C:273, F:635, N:899	0.85	0.43	0.56–1.28	0.99	0.93	0.81–1.22	0.86	0.45	0.58–1.27
Distant metastasis C:292, F:681, N:951	1.11	0.81	0.48–2.54	0.83	0.35	0.57–1.22	0.78	0.5	0.38–1.60
Prostate cancer mortality C: 287, F:667, N:931	0.87	0.9	0.11–6.95	0.87	0.78	0.34–2.26	0.66	0.62	0.12–3.47
Nonprostate cancer mortality C: 287, F:667, N:931	2.34	0.001	1.40–3.89	1.87	0	1.42–2.47	2.65	0	1.68–4.18
Overall mortality C: 289, F:670, N:934	1.95	0.005	1.22–3.11	1.62	0	1.27–2.07	2.07	0.001	1.36–3.14
Cox proportional hazards at 3 years (3 PrCa deaths, 22 deaths from any cause)									
Prostate cancer mortality C: 187, F:479, N:625	0.88	0.8	0.34–2.30	0.66	0.63	0.12–3.51	2.34	0.001	1.41–3.90
Nonprostate cancer mortality C: 187, F:479, N:625	1.88	0	1.42–2.47	2.66	0	1.68–4.20	1.96	0.005	1.23–3.12
Overall mortality C: 189, F:482, N:628	1.63	0	1.28–2.07	2.07	0.001	1.36–3.15	2.07	0.001	1.36–3.15
Cox proportional hazards at 5 years (5 PrCa deaths, 34 deaths from any cause)									
Prostate cancer mortality C: 145, F:372, N:427	0.85	0.88	0.10–6.97	0.88	0.79	0.34–2.29	0.64	0.6	0.12–3.41
Nonprostate cancer mortality C: 145, F:372, N:427	2.38	0.001	1.43–3.98	1.85	0	1.41–2.44	2.65	0	1.68–4.20
Overall mortality C: 147, F:375, N:430	1.97	0.004	1.23–3.15	1.61	0	1.26–2.05	2.05	0.001	1.35–3.12
Cox proportional hazards at 10 years (120 biochemical failure, 148 treatment failure, 24 distant metastasis, 13 PrCa deaths, 74 deaths from any cause)									
Prostate cancer mortality C: 78, F:174, N:160	0.8	0.85	0.08–8.00	0.83	0.71	0.31–2.22	0.61	0.58	0.10–3.60
Nonprostate cancer mortality C: 78, F:174, N:160	2.17	0.003	1.30–3.63	1.66	0	1.26–2.19	2.31	0	1.46–3.65
Overall mortality C: 80, F:177, N:163	1.79	0.016	1.12–2.86	1.45	0.003	1.14–1.86	1.8	0.006	1.18–2.74

F, Former smoker; N, Never smoker.

a C, Current smoker

**Figure 1:**
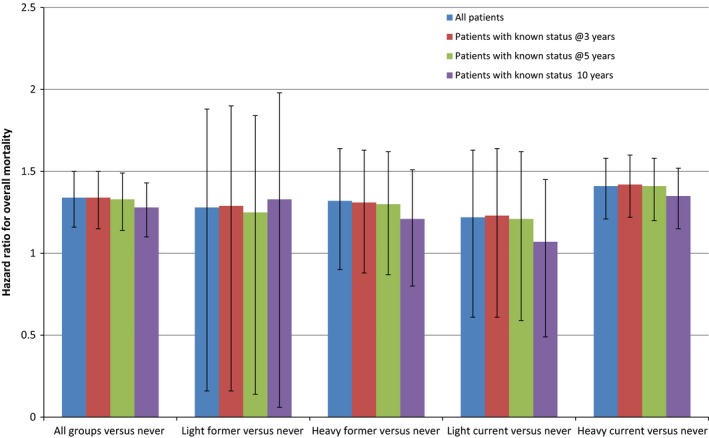
Hazard ratios for overall mortality of light former, heavy former, light current, and heavy current smokers compared to never smokers.

Figure 1 is based on proportional mortality hazards analysis, comparing never smokers to four smoking categories: current light smokers, current heavy smokers, former light smokers, and former heavy smokers. Heavy smokers are those with more than 25 pack years; light smokers are those with 25 or fewer pack years. The hazards analyses for the 3‐, 5‐, and 10‐year follow‐up periods are based on subjects with complete data for each period. The analysis for the entire follow‐up period has subjects lost to follow‐up censored at the time they were lost to follow‐up. The “light” and “heavy” classifications for former and current smokers are based on the pack years smoked: heavy smokers smoked more than the median pack years and light smokers smoked fewer. It can be seen that the proportional hazards ratios tend to be highest for current heavy smokers, although the differences between this category and other categories of smoker are not statistically significant. The proportional hazards for the 10‐year follow‐up period tend for current smokers to be lower than those for shorter periods; this is likely due to the fact that many of the current smokers quit smoking during the extended follow‐up period. Also, although the point estimates of the proportional hazards associated with smoking status are statistically significant only for heavy current smokers, and for all smokers combined, all these point estimates are positive. Because pack years could be closely correlated with age, we reran the analyses, dropping age as a control variable; it made no difference to the findings.

## Discussion

Given the well‐known impact of smoking upon overall life expectancy, the substantial association of smoking with survival of CaP patients after RP is not surprising. Nonetheless, it is worth noting and emphasizing that smoking among patients with prostatectomy‐treated CaP puts them at statistically significant, increased overall mortality risk: greater risk at 3, 5, 10 years, and longer periods of time. CaP patients with operable disease deserve to be informed that they are several times more likely to die of other causes than of CaP and that smoking at diagnosis is associated with a statistically and substantively significant increase in immediate and long‐term mortality risk.

The association of smoking with increased overall mortality in these patients with operable CaP does not appear to be mediated by CaP‐specific outcomes—biochemical failure, treatment failure, distant metastasis, or disease‐specific mortality. Overall mortality showed a significantly higher overall hazards ratio and 3‐, 5‐, and 10‐year hazards ratios for current smokers compared to former smokers or never smokers. When pack years smoked was used to further classify former and current smokers as either light or heavy in terms of exposure, overall mortality showed a significantly higher hazards ratio and 3‐, 5‐, and 10‐year odds ratios for heavy current smokers compared to never smokers. Although current smokers had a higher diagnostic PSA, and were more likely to have lymph node metastases and positive surgical margins, smoking was unrelated to treatment failure, presence of distant metastasis, or disease‐specific mortality in any analysis with these factors taken into account. However, the power to detect differences for this sample of patients was limited. Overall mortality for CaP patients who undergo RP is not heavily dependent on CaP‐specific outcomes; among these patients, only 13 of the 74 patients who died within 10 years died of CaP. Similarly, Kenfield's analysis (11) of prostate cancer outcomes revealed that only 524 of 1630 patients died of CaP, while over 1100 died of other causes.

It would have been valuable to evaluate years since smoking cessation: unfortunately, separating former smokers according to the years since smoking complicated the analysis and lessened statistical precision. We experimented with interaction terms representing the combined association of pack years and years since smoking; the findings did not change substantively.

A small number of studies have suggested that smoking increases risk of CaP aggressiveness 3, 7, 8. The results of this analysis for patients with operable disease showed smoking to be weakly or negligibly associated with aggressiveness, but strongly associated with overall mortality. Since the implementation of PSA testing for early detection of CaP, many patients diagnosed with CaP die of other comorbidities—typically cardiovascular disease 9, 10. Bittner et al. 2. determined that the leading cause of death for CaP patients treated with brachytherapy was cardiovascular disease. Kenfield et al. 11. showed significantly increased age and multivariate‐adjusted CaP mortality in current smokers, and a significantly higher hazard of overall mortality, independent of covariates used, for former and current smokers.

Of the previous studies that examined the association between smoking and CaP recurrence after primary treatment (including RP 12, 13, 14, external beam radiation 15, 16, or brachytherapy 17), all but one study 17 found evidence for increased aggressiveness of CaP associated with smoking history. Whether they adequately addressed confounding in these aggressiveness analyses can be argued. Three of these studies 12, 15, 16 examined mortality and metastasis outcomes separately; they all reported a significantly higher hazard of overall mortality for smokers. None found statistically significant alteration of risk for CaP‐specific mortality, although this may be due to a small number of events. The conclusions of these studies were inconsistent regarding the association of smoking with metastasis. Moriera et al. 12. found that current smokers had a significantly higher hazard of metastasis than never or former smokers; however, the small number of patients with metastasis (26 [1.31%]) resulted in a large confidence interval (HR: 2.51, 95% CI: 1.03–6.11). Pantarotto et al. 15. found that smokers were not significantly more likely to have local failure or biochemical failure after external beam radiation therapy, but that smokers were significantly more likely to experience distant failure. Pickles et al. 16. found smoking to be associated with increased risk of metastasis after external beam radiation therapy, but rates were very low. While all studies showed an elevated hazard of mortality among smokers, the conflicting results for metastatic disease highlight the controversy surrounding smoking and CaP aggressiveness.

The limitations of this study include that smoking history was collected at the time of RP and not followed for changes. This inability to document smoking subsequent to diagnosis and treatment in all likelihood has resulted in an underestimate of the impact of smoking at diagnosis. Joshu et al. 14. found that although no former or never smokers began smoking at 1 year after RP, 46% of patients who were smokers 5 years before RP had quit by 1 year after RP. Thus, some of the patients categorized as current smokers at RP quit during follow‐up. Some patients categorized as never or former smokers may have misrepresented their smoking status, when smoking history was collected after CaP diagnosis. This study was not designed to adjust for exposure to second‐hand smoke or puffing patterns, both of which can affect total tobacco exposure. Similar to earlier studies, the low number of patients with distant metastasis (72 [4%]) and CaP‐specific mortality (21 [1%]) limits the ability to determine the association of risk with these outcomes. These patients, all with operable disease, were treated at a single institution, and who all underwent RP, represent a relatively homogeneous group. Homogeneity is probably a strength of this study, while heterogeneity makes it more difficult to adjust for the mélange of factors that might affect outcome. In Kenfield et al. 11, for example, smokers were more likely than never smokers or those who had quit 10 or more years prior to diagnosis to be diagnosed with advanced disease, with higher Gleason grade, to be treated with hormones or watchful waiting, to have an elevated PSA at diagnosis; they were less likely to be treated by prostatectomy and less likely to have been diagnosed by PSA. Inability to precisely measure such confounders increases the likelihood of residual confounding 18, 19. The homogeneity of this study is an advantage, speaking to the diminished likelihood of confounding due to the stage or grade of disease, hospital treatment patterns, or access. Other confounders, though, are clearly possible. In addition, these results might not describe the dynamics and effects of smoking among other populations of CaP patients.

Any therapeutic factor associated with a greater than twofold alteration of mortality risk—as smoking at CaP diagnosis is—deserves attention. Although oncology practitioners almost universally acknowledge that smoking among cancer patients diminishes their chance for surviving their disease, too few expend more than minimal effort in encouraging smoking cessation 20. The finding of this study—smoking is associated with substantially increased overall mortality among CaP patients treated with RP—supports the importance of physician recommendation and/or implementation of smoking cessation programs by physicians who treat CaP patients.

## Conclusions

This study confirms that smoking is strongly associated with overall mortality among CaP patients with operable disease, who can expect to live for several years after RP. The association of smoking with overall mortality risk is comparable to or stronger than its association with CaP progression, whether defined by biochemical failure, treatment failure, or development of metastatic disease. This finding and similar findings in the literature mandate the need for recommendations and/or implementation of smoking cessation programs by physicians who treat CaP patients and greater use of existing programs by CaP patients.

## Conflict of Interest

The authors have no conflict of interest to disclose.
